# Common Diagnostic Challenges and Pitfalls in Segmental Colitis Associated with Diverticulosis (SCAD)

**DOI:** 10.3390/jcm12186084

**Published:** 2023-09-20

**Authors:** Caterina Sbarigia, Camilla Ritieni, Bruno Annibale, Marilia Carabotti

**Affiliations:** Department of Medical-Surgical Sciences and Translational Medicine, Sapienza University, 00189 Rome, Italy; cate.sbarigia@gmail.com (C.S.); camilla.ritieni@gmail.com (C.R.); bruno.annibale@uniroma1.it (B.A.)

**Keywords:** diverticular disease, segmental colitis associated with diverticulosis, haematochezia, drug-inducing colitis, biopsies

## Abstract

Segmental colitis associated with diverticulosis (SCAD) is characterized by inflammation involving the sigmoid inter-diverticular mucosa, sparing the proximal colon and rectum. Due to the heterogeneity of clinical manifestations and endoscopic and histological findings, SCAD diagnosis might be challenging in clinical practice. This narrative review aimed to report the SCAD diagnostic criteria adopted in different studies, highlighting the current challenges and main pitfalls in its diagnosis. We analysed fourteen studies, mainly prospective observational studies. Haematochezia and rectal bleeding were the main complaints leading to diagnosis, followed by diarrhoea. An accurate endoscopic description was performed in 86% of studies, while a standardised biopsy sampling protocol (sigma, proximal colon and rectum) was scarcely adopted, being complete only in 28.5% of studies. The evaluation of concomitant drugs potentially inducing colitis was carried out in only 57% of studies. Great heterogeneity in sigmoid endoscopic (edema, erythema, erosions, ulcers, mucosal friability) and histological findings (chronic and/or acute inflammatory infiltrate) was observed. We showed that SCAD diagnosis is often based on not fully adequate macroscopic colonic description and scant biopsy protocol sampling. An accurate clinical and endoscopic evaluation, with an adequate sampling biopsy protocol, with attention to differential diagnosis, seemed to be crucial for a prompt SCAD diagnosis.

## 1. Background

Diverticular disease is a frequent condition in Western countries, affecting up to two-thirds of people older than 80 years [1]. The clinical spectrum of diverticular disease is very heterogenous and includes various conditions. The majority of subjects with colonic diverticula remain asymptomatic throughout life (i.e., diverticulosis), approximately 15–20% will develop abdominal symptoms as pain eventually associated to alterations in bowel habits or bloating (i.e., symptomatic uncomplicated diverticular disease) and about 5% develop complications, including acute diverticulitis or diverticular bleeding [2,3,4]. Among diverticular complications, another separate clinical entity has been described, known as segmental colitis associated with diverticulosis (SCAD).

SCAD was initially described in the early 1980s in a small cohort of patients as a form of colitis characterized by sigmoid diverticulosis with mucosal inflammation confined to the segment of the colon bearing the diverticula [5,6]. The exact prevalence of SCAD is not well determined, varying in different cohorts from 1.9 to 11% of patients with diverticulosis [7,8,9].

SCAD involves patients who are typically male and over 60 years of age [8,9,10,11]. Clinical manifestations are heterogeneous, including haematochezia or intermittent rectal bleeding, lower abdominal pain and alterations to bowel habits, mainly diarrhoea [2,7,8,12]. Sometimes, SCAD diagnosis is incidental during routine endoscopy for colon rectal cancer screening [13,14]. Systemic symptoms and/or signs, like fever, nausea, vomiting and weight loss, are rarely reported [15,16].

Endoscopic examination in SCAD patients shows an inflammatory process restricted to the sigma, characterized by a wide range of findings, from oedema and erythema of the inter-diverticular mucosa to the presence of erosions, mucosal friability and ulcers [2]. The spectrum of histological findings is also very variable, from unspecific chronic or acute inflammation signs to inflammatory bowel disease (IBD)-like alterations. The rectum and proximal colon are always spared, both at endoscopic and histological evaluation [2,7,16]. 

Usually, SCAD diagnosis is based on endoscopic and histological evidence of inflammation limited to the inter-diverticula mucosa, but a standardized biopsy protocol, including rectum and proximal colon sampling, is rarely performed. In addition, SCAD features might be difficult to distinguish from other colitis, such as IBD, drug-induced colitis and infective or ischemic colitis, requiring an accurate evaluation for a correct differential diagnosis [17,18].

Due to the heterogeneity in clinical manifestations and endoscopic and histological findings, SCAD diagnosis might be challenging in clinical practice. 

The aim of this narrative review is to report the SCAD diagnostic criteria adopted in different studies, highlighting the current challenges and main pitfalls in its diagnosis.

## 2. Materials and Methods

This narrative review describes SCAD diagnostic criteria adopted in the current literature. The SANRA scale was used to structure the present narrative review [19]. A literature search was conducted using Pubmed until 1 August 2023. Original articles were identified using the following search terms: “segmental colitis associated with diverticulosis”, “segmental colitis associated to colonic diverticula”. Reviews, abstracts, letters to the editor and case reports were excluded. Additional articles were identified by reviewing the reference sections of all selected articles. 

## 3. Results

The characteristics of the selected studies, including type of study, number of patients, gender, age and abdominal symptoms and/or signs leading to SCAD diagnosis, are reported in Table 1. 

We analysed fourteen studies, mainly prospective observational (78.6%), including a small number of patients, ranging from 10 to 129. Patients were mostly male (59%), with their ages ranging from 50 to 73 years old. 

Regarding clinical presentation, haematochezia and rectal bleeding were the main abdominal symptoms/signs leading to diagnosis, being present in 76–93% of patients, followed by diarrhoea, present in 44–86%. Abdominal pain was less frequently reported. In two studies, patients’ symptoms or signs were not reported at all.

**Figure 1:**
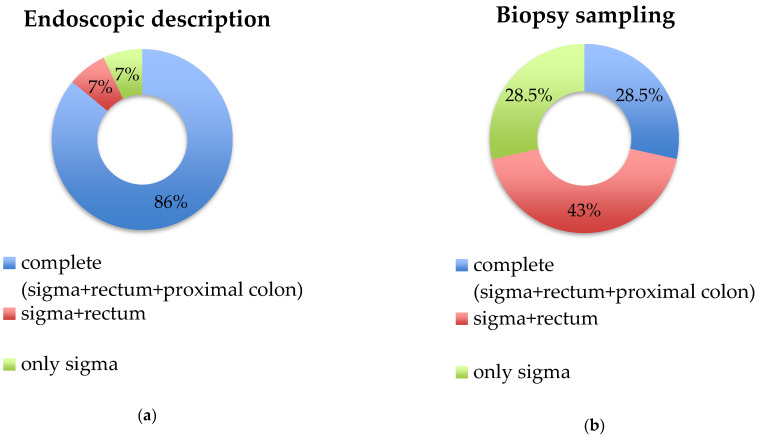
Endoscopic description and biopsy sampling in the included studies. (**a**) Endoscopic description; (**b**) biopsy sampling. Legend: for each study, the biopsy sampling group was assigned to the best category only if the protocol had been applied in ≥60% of patients of the study.

As shown in Figure 1a, a complete endoscopic description of the sigma, rectum and proximal colon was assessed in twelve out of the fourteen studies (86%), whereas in the remaining two studies, the description was partial, lacking the rectum and/or proximal colon [11,20].

Table 2 reports SCAD endoscopic findings for the sigma, proximal colon and rectum, and whether an evaluation of concomitant drugs potentially inducing colitis was carried out. The most common sigmoid endoscopic findings were oedema and erythema (12 out of 14 studies, 86%), followed by erosions and/or ulcers and mucosal friability (11 out of 14 studies, 78%); unspecific signs of inflammation were poorly reported (7%). Oedema, erosions and friability were reported together in 71% of the studies. In 2010, a SCAD endoscopic classification was proposed by identifying four different subtypes: pattern A, “crescentic fold disease” pattern (swollen red patches from 0.5 to 1.5 cm in diameter of colonic mucosa without ulcerations or signs of bleeding confined to the crescentic mucosal folds with sparing of diverticular orifices); pattern B, “mild-to moderate ulcerative colitis-like” pattern (diffuse loss of vascular pattern, mucosal oedema and hyperaemia and erosions, similar to mild to moderate ulcerative colitis but with sparing of diverticular orifices); pattern C, “Crohn’s disease colitis-like” pattern (scattered aphthous ulcers within a normal colonic mucosa, with normal vascular pattern, sparing of diverticular orifices, like a mild-to-moderate Crohn’s colitis); pattern D, ”severe ulcerative colitis-like” pattern (diffuse loss of vascular pattern, hyperaemia and contact bleeding, marked mucosal oedema with ulceration resembling a severe ulcerative colitis) [7]. Of note, in a study by Gore et al., the endoscopic description was unclear since a some patients did not present colonic diverticula (18%) [20]. In addition, in a study by Koutrobakis et al., 8.7% of patients presented unspecified lesions in the right colon [8].

The concomitant use of drug-inducing colitis was evaluated in only eight studies (57%). Specifically, the recent use of NSAIDs was considered, and in two studies, the use of COXIB was also reported.

As shown in Figure 1b, biopsy sampling was complete (sigma, rectum and proximal colon) only in four studies (28.5%). Biopsies of the rectum and sigma were collected in six studies (43%) [8,20,21,22,23,24], while the remaining four studies collected only sigma samples (28.5%) [10,11,14,15]. Details regarding biopsy protocol sampling are reported in Table 3. Of note, in some studies, biopsy protocol sampling differed between patients; specifically, in a study by Makapugay et al., biopsy samples of the rectum and proximal colon were performed in 8.7% and 21.7% of patients, respectively [10], while Ierardi and colleagues collected sigma biopsies but not in all patients diagnosed as SCAD (92.8%) [11]. Finally, in a recent paper by Vulsteke et al., rectum biopsies were taken only in nineteen out of thirty-seven patients (51%) [14].

The main histopathological criteria utilized to diagnose SCAD are reported in Table 3. Chronic inflammatory infiltrate, acute inflammatory infiltrate and cryptits/crypt abscesses were the histopatological findings most frequently reported (57%), followed by goblet cell depletion (50%), crypt distortion and/or atrophy (28%) and unspecific inflammation (14%). In a study by Gore at al. [20], rectum sparing was not present in 18% of patients.

## 4. Discussion

SCAD is a pathological entity characterized by an inflammatory response involving the sigmoid inter-diverticular mucosa. The rectum and the right colon are always spared from inflammation. Limitations of the mucosal lesion to the diverticular segment are the most important criterion for SCAD diagnosis, so rectal and descending colon biopsies are required to distinguish SCAD from other colitis (i.e., IBD, drug-induced colitis, ischemic colitis, infective colitis). 

In this review, we reported the SCAD diagnostic criteria adopted in different studies to highlight the current challenges and pitfalls in its diagnosis. 

We found that an accurate endoscopic description was provided in about 85% of studies, while a standardised biopsy sampling protocol (sigma, proximal colon and rectum) has been scarcely adopted, being complete in only 28.5% of studies. Specifically, regarding the endoscopic description, in about 15% of studies, an assessment of the rectum and/or proximal colon was lacking. Gore et al. did not describe the mucosal appearance of the descending colon [20], whereas Ierardi et al. did not report the endoscopic findings of either the descending colon or rectum [11]. 

Regarding biopsy sampling, surprisingly, we showed that a complete biopsy protocol, including sigma, rectum and proximal colon, was performed in only about 30% of cases. In the remaining studies, the protocol sampling was partial, with biopsies performed only in sigma in 28.5% [10,11,14,15] and in both the sigma and rectum in 43% of cases [8,20,21,22,23,24]. All these results highlighted how SCAD diagnosis is often based on a macroscopic description of the colon, sometimes even not fully adequate, and scant biopsy protocol adherence, potentially leading to the risk of misdiagnoses, unnecessary endoscopic examinations and chronic therapies.

Regarding endoscopic findings, we found great heterogeneity in sigmoid lesions, ranging from oedema and erythema to erosions, ulcers, mucosal friability or unspecific signs of inflammation. The heterogeneity in endoscopic findings could make the diagnosis of SCAD challenging, since, sometimes, there are very mild or unspecific endoscopic lesions that can be poorly recognizable. Furthermore, we highlighted how, sometimes, the endoscopic findings are not fully compatible with SCAD diagnosis. In the study of Gore et al., 18% of patients did not present colonic diverticula, raising questions regarding the reliability of the diagnosis [20]. In addition, in a retrospective study by Koutroubakis et al., 9% of patients presented the endoscopic unspecified involvement of the proximal colon [8]. The authors did not better describe the characteristics of these lesions, nor did they perform biopsies. Again, the endoscopic features, which are considered to be SCAD, do not appear fully consistent with the diagnosis. Of note, Tursi et al. proposed a SCAD endoscopic classification, distinguishing four patterns of endoscopic sigmoid findings and including injuries ranging from milder (type A and C) to more severe damage (type B and D) [7,23]. However, this classification is not widespread and is rarely used. 

We observed great heterogeneity in histological lesions too, including chronic inflammatory infiltrate, acute inflammatory infiltrate, crypt distortion or atrophy, cryptitis or crypt abscesses, goblet cell depletion and even just unspecific inflammation. In a prospective study published in 2010 by Tursi et al., the authors adopted a SCAD histological classification, considering three subtypes of histological findings: ulcerative colitis-like picture (cryptic abscesses or glandular architectural distortion); Crohn’s disease-like picture (full mucosal thickness, inflammatory infiltrate); nonspecific colitis without specific features of ulcerative colitis or Crohn’s disease [7]. In later studies, Tursi et al. evaluated colonic specimens through two scores: the first aimed to assess the mucosal damage and the second the inflammation activity. Mucosal damage was assessed considering different histological lesions (i.e., polymorphonuclear infiltration of the epithelium and lamina propria, crypt abscesses, loss of glandular parallelism, crypt shortening and/or ramification, mucus epithelial depletion and involvement of muscularis mucosae and/or submucosa), whilst inflammation activity was expressed by the total number of neutrophils in the lamina propria. Each of these histological lesions was measured using a score from 0 to 3 [21,22,25]. Again, this histological score is poorly used, and it is adopted only in a few studies. Finally, we underlined that, in a study by Gore et al., rectum involvement was reported in 9% of patients, with the presence of active inflammation with crypt architectural changes and alterations of goblet cells [20], raising doubts that it could be inflammatory bowel disease, possibly ulcerative colitis. These data further emphasized how the SCAD diagnosis might be challenging and, sometimes, not fully adequate. 

Currently, knowledge of this condition is scarce, and only a few guidelines concerning diverticular disease refer to SCAD diagnosis, probably because of the uncertain prevalence of this condition [2,26]. Specifically, the Italian consensus conference for colonic diverticulosis and diverticular disease, published in 2014, provided four statements concerning SCAD, considering its definition, epidemiology, clinical manifestations, diagnosis and therapies. The authors stated that rectal and descending colon biopsies are always required to make a prompt SCAD diagnosis. Finally, the latest 2022 German diverticular disease guidelines have one brief statement regarding SCAD, assessing that diverticulosis can be associated with segmental colitis. However, no statements considering diagnostic criteria are reported. 

In an observational study aiming to assess SCAD prevalence, less than 5% of diverticulosis patients were identified as having suspected SCAD, but none of these patients were confirmed via histological examination. Adopting a complete biopsy protocol, the authors found that one suspected SCAD patient had a new diagnosis of Crohn’s disease, one patient presented lesions compatible with intestinal spirochetosis and one presented with drug-induced colitis due to NSAID use, highlighting that standardized biopsy sampling helps to make a correct diagnosis, preventing overdiagnosis and useless therapies [27].

As is known, SCAD damage is restricted to the interdiverticular mucosa; the role of endoscopic evaluation together with prompt histological sampling is crucial for its diagnosis. Differently from other complications of diverticular disease, during which the muscle layers and/or the colonic and pericolic tissue may be involved (i.e., acute diverticulitis, stenosis and perforation), in SCAD diagnosis, the role of radiology is very limited. Particularly, abdomen-contrasted enhanced CT scan should be considered only in the case of the coexistence of significant bleeding or new appearance or worsening of constipation, suggesting colonic stenosis. 

A further important issue is the differential diagnosis with other colitis, i.e., IBD, drug-induced colitis, ischemic colitis and infectious colitis. With respect to IBD, some endoscopic findings in SCAD may be similar to common features reported in ulcerative colitis or Crohn’s disease. In fact, whereas the main endoscopic lesions in SCAD patients are oedema and erythema, the diffuse loss of vascular pattern, erosions, contact bleeding, ulcers and scattered aphthous ulcers have also been described, which are typical IBD endoscopic patterns. Furthermore, the histological lesions described in SCAD can range from mild unspecific inflammation to florid acute or chronic inflammatory changes, mimicking ulcerative colitis or Crohn’s disease and including cryptic abscesses, glandular architectural distortion, inflammatory infiltrate in the lamina propria, lymphoid follicles and goblet cell depletion [28]. Even if there are a lot of common and misleading aspects, it is worth highlighting that SCAD never affects the rectum nor proximal colon, both macroscopically and histologically, distinguishing itself from ulcerative colitis and Crohn’s disease. This point underlines that a complete endoscopic and histological evaluation, including the rectum and proximal colon, is mandatory for a proper differential diagnosis. It is essential to make a distinction between these two conditions, SCAD and IBD, since the natural history and prognosis vary significantly. In SCAD, long-term medications are often not required, whereas it is known that IBD patients need lifelong therapies. To make things more complicated, it has been reported that SCAD may sometimes precede the onset of ulcerative colitis with rectal involvement, although this progression has been described only in case reports [29,30]. 

It is known that a lot of different drugs can injure the colonic mucosa, mimicking SCAD damage. Drug-induced colitis may be described following treatment with NSAIDs, laxatives, diuretics, carbamazepine, isotretinoin, mycophenolate mofetil, kayexalate, oral contraceptives, substances of abuse (i.e., cocaine and amphetamines) and, finally, most recent biological agents acting via inhibition of key regulatory molecules, such as brentuximab, ipilimumab, rituximab and etanercept. The patterns of endoscopic and histological injury in drug-induced colitis are various, ranging from mild to severe lesions and including unspecific findings [31]. We reported that less than 60% of the included studies examined drugs potentially responsible for colitis, considering just NSAIDs and COXIB. Since differential diagnosis is based on clinical suspicion, it appears to be critical to carefully investigate concomitant drugs potentially injuring the gastrointestinal tract, considering dosages and the temporal relationship. We underlined the need to meet specific diagnostic SCAD criteria, since other conditions (i.e., IBD, drug-induced colitis, ischemic colitis, infectious colitis) have different therapeutic management and follow-up.

## 5. Conclusions

This review shows that SCAD diagnosis is often based on macroscopic colonic description that is not fully adequate and scant biopsy protocol sampling. The wide heterogeneity in endoscopic and histological lesions, together with the necessity of a prompt differential diagnosis with other colitis, further contributes to the increased challenges in its diagnosis. An accurate anamnestic and clinical evaluation, as well as a careful endoscopic examination with an adequate sampling biopsy protocol, seems to be crucial for a prompt SCAD diagnosis.

## Figures and Tables

**Table 1:** Main characteristics of included studies and segmental colitis associated with diverticulosis (SCAD) population. Legend: PTS, patients; SCAD, segmental colitis associated diverticulosis.

Author, Year	Type of Study	N° of pts	Male (%)	Mean Age (Years)	Main Abdominal Symptoms and/or Signs	Other Symptoms and/or Signs
Gore, 1992 [20]	prospective observational	34	71	60	Rectal bleeding (77%)	Diarrhoea and abdominal pain (lower left quadrant)
Makapugay, 1996 [10]	prospective observational	23	52	72	Intermittent haematochezia (83%)	Rectal bleeding and lower abdominal pain; nonlocalized abdominal pain without blood loss; concomitant chronic constipation; episodic diarrhoea
Koutroubakis, 2005 [8]	retrospective observational	23	74	62	Haematochezia (78%)	Abdominal pain (61%), diarrhoea (39%)
Imperiali, 2006 [15]	prospective observational	15	60	63	Haematochezia (93%)	Abdominal pain or fever (20%)
Freeman, 2008 [12]	prospective observational	24	58	50	Diarrhoea (75%)	Bloody stools (71%), abdominal pain (67%)
Ierardi, 2008 [11]	prospective observational	14	57	66	Haematochezia (93%)	Diarrhoea (50%), abdominal pain (36%)
Tursi, 2010 [7]	prospective observational	92	46	62	Chronic diarrhoea (46%)	Recurrent abdominal pain (11%), rectal bleeding (10%)
Tursi, 2011 [21]	prospective observational	21	71	59	Not reported	Not reported
Tursi, 2011 [22]	prospective observational	10	60	59	Not reported	Not reported
Tursi, 2011 [23]	retrospective observational	129	60	64	Chronic diarrhoea (45%)	Recurrent abdominal pain (12%), recurrent rectal bleeding (15%)
Tursi, 2011 [24]	prospective observational	27	44	64	Chronic diarrhoea (44%)	Recurrent abdominal pain (11%) recurrent rectal bleeding (7%)
Tursi, 2012 [25]	prospective observational	27	48	62	Chronic diarrhoea (44%)	Recurrent abdominal pain (11%) and recurrent rectal bleeding (11%)
Vulsteke, 2021 [14]	retrospective observational	37	57	73	Haematochezia (76%)	Diarrhoea (24%), abdominal pain (35%)
Tursi, 2023 [9]	prospective observational	44	68	64	Diarrhoea (86%)	Not reported

**Table 2:** Endoscopic features and evaluation of concomitant drugs potentially inducing colitis of the included studies. When ‘X’ is reported means that the finding was present or the assessment was provided. Legend: PTS, patients.

Author, Year	Endoscopic Findings Sigma				Endoscopic Findings Proximal Colon	Endoscopic Findings Rectum	Evaluation of Drugs Potentially Inducing Colitis
	Oedema/Erythema	Erosions/Ulcers	Friability	Unspecific Inflammation			
Gore, 1992 [20]	X				not reported	spared	
Makapugay, 1996 [10]	X		X		spared	spared	
Koutroubakis, 2005 [8]	X	X	X		spared (in 21/23 pts)	spared	X
Imperiali, 2006 [15]		X			spared	spared	X
Freeman, 2008 [12]				X	spared	spared	X
Ierardi, 2008 [11]	X	X	X		not reported	not reported	X
Tursi, 2010 [7]	X	X	X		spared	spared	
Tursi, 2011 [21]	X	X	X		spared	spared	X
Tursi, 2011 [22]	X	X	X		spared	spared	
Tursi, 2011 [23]	X	X	X		spared	spared	
Tursi, 2011 [24]	X	X	X		spared	spared	X
Tursi, 2012 [25]	X	X	X		spared	spared	
Vulsteke, 2021 [14]	X	X	X		spared	spared	X
Tursi, 2023 [9]	X	X	X		spared	spared	X

**Table 3:** Biopsy protocol and histological findings of the included studies. Legend: PTS, patients; NA, not applicable. When ‘X’ is reported means that the finding was present or the assessment was provided.

Author, Year	Biopsy Sigma	Biopsy Proximal Colon	Biopsy Rectum	Histological Findings Sigma						Histological Findings Proximal Colon	Histological Findings Rectum
				Chronic Inflammatory Infiltrate	Acute Inflammatory Infiltrate	Crypt Distortion/ Atrophy	Cryptitis/Crypt Abscesses	Goblet Cell Depletion	Unspecific Inflammation		
Gore, 1992 [20]	yes	not	yes	X	X		X	X		NA	spared in 91%
Makapugay, 1996 [10]	yes	not (in 21/23 pts)	not (in 18/23 pts)	X		X	X			spared	spared
Koutroubakis, 2005 [8]	yes	not	yes	X	X	X	X	X		NA	spared
Imperiali, 2006 [15]	yes	not	not	X	X					NA	NA
Freeman, 2008 [12]	yes	yes	yes	X				X		spared	spared
Ierardi, 2008 [11]	yes (in 13/14 pts)	not	not		X		X			NA	NA
Tursi, 2010 [7]	yes	yes	yes	X	X			X		spared	spared
Tursi, 2011 [21]	yes	not	yes					X		NA	spared
Tursi, 2011 [22]	yes	not	yes		X		X			NA	spared
Tursi, 2011 [23]	yes	yes	yes						X	spared	spared
Tursi, 2011 [24]	yes	not	yes						X	NA	spared
Tursi, 2012 [25]	yes	not	yes		X		X			NA	spared
Vulsteke, 2021 [14]	yes	not	not (in 18/37 pts)	X		X	X	X		NA	spared (when performed)
Tursi, 2023 [9]	yes	yes	yes	X	X	X	X	X		spared	spared

## Data Availability

Data sharing not applicable.
